# Supplementary material for: Model-free inference of direct network interactions from nonlinear collective dynamics
Source: Nat Commun. 2017 Dec 19;8:2192. doi: 10.1038/s41467-017-02288-4 (PMC5736722; doi:10.1038/s41467-017-02288-4)
Supplement: Supplementary file 1 — Supplementary Information [file 41467_2017_2288_MOESM1_ESM.pdf]

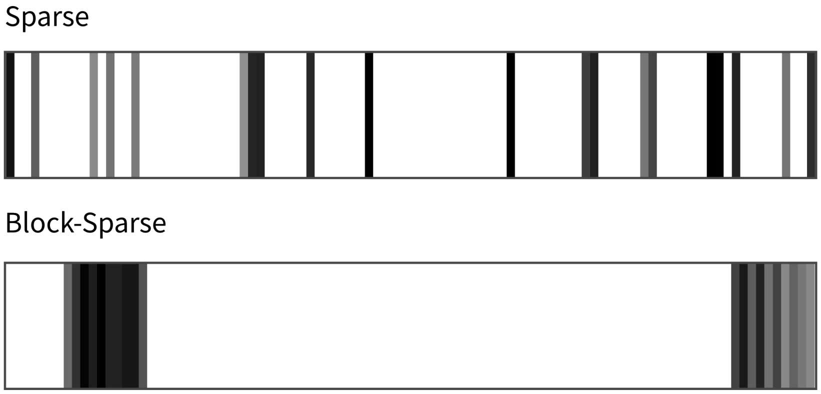

Supplementary Figure 1: **Sparsity differs from block sparsity.** A sparse vector contains only few entries different that zero distributed along the vector. Instead, a block sparse vector is composed by few blocks of non-zero entries.

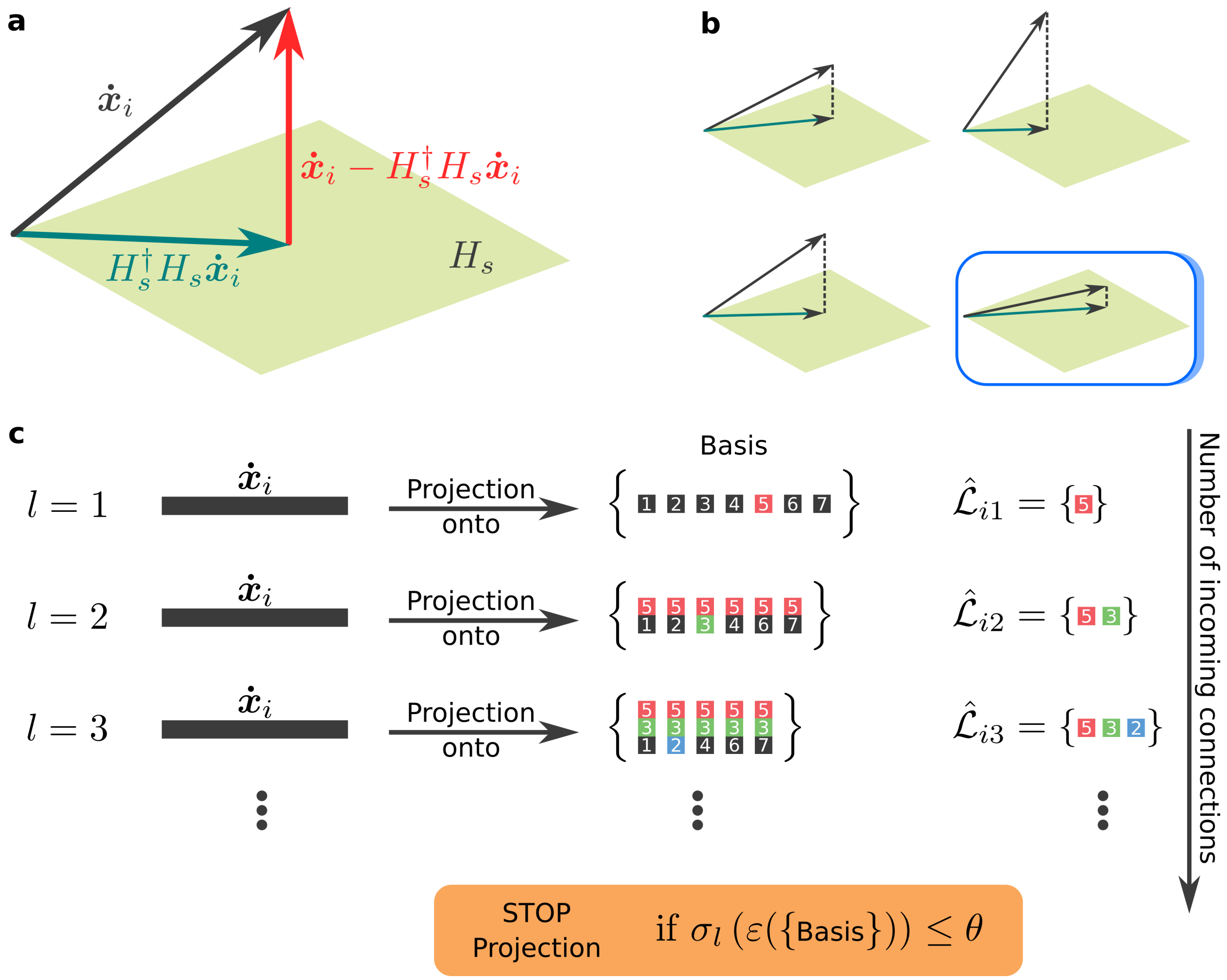

Supplementary Figure 2: **ARNI reveals connections by projecting dynamics onto spaces spanned by basis functions.** **a**, ARNI projects dynamical vectors $\dot{\boldsymbol{x}}_i$ onto spaces spanned by H_s, where $H_s^\dagger H_s \dot{\boldsymbol{x}}_i$ is the projection and $\dot{\boldsymbol{x}}_i - H_s^\dagger H_s \dot{\boldsymbol{x}}_i$ the error of the projection. **b**, ARNI selects the H_s yielding the largest projection, or conversely, the smallest error. **c**, ARNI iteratively constructs $\hat{\mathcal{L}}_i$ by means of $\hat{\mathcal{L}}_{i,l}$. At $l = 1$, $\dot{\boldsymbol{x}}_i$ is projected on all spaces and one incoming interaction n^* (red) is selected according to (12). At $l = 2$, $\dot{\boldsymbol{x}}_i$ is now projected on the composite spaces formed by the inferred incoming connection in $\hat{\mathcal{L}}_{i,1}$ and the rest of possible incoming interactions in $\mathcal{C}_1$. Then, one incoming interaction n^* (green) is selected according to (12). At $l = 3$, $\dot{\boldsymbol{x}}_i$ is projected in the composite spaces formed by the inferred incoming interactions in $\hat{\mathcal{L}}_{i,2}$ and the rest of possible incoming interactions in $\mathcal{C}_2$. Then, an incoming interaction n^* (blue) is selected according to (12). This process is repeated until the stopping criterion $\sigma_l \leq \theta$ is reached.

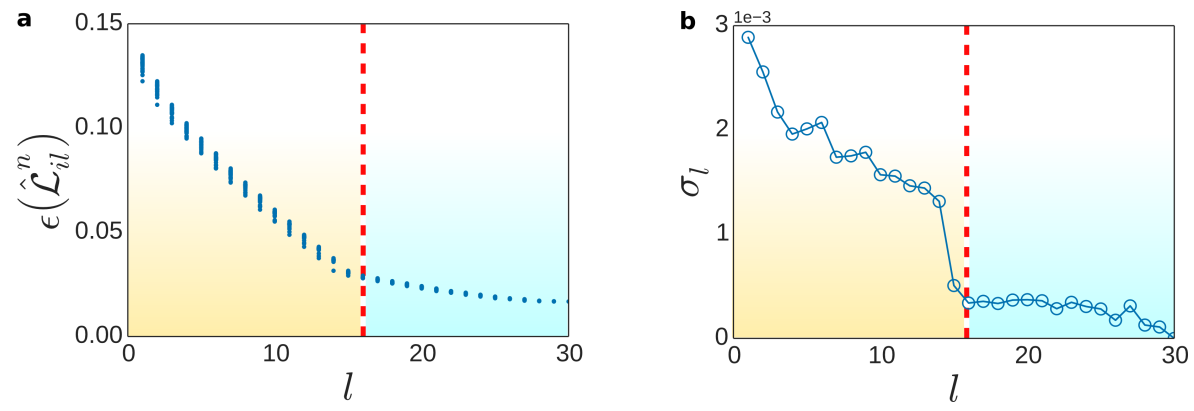

Supplementary Figure 3: **ARNI iteratively arrives at the right solution.** Reconstruction of a selected unit of a Kuramoto-like oscillators (17) of $N = 30$ and $n_i = 15$. **a**, Error function $\epsilon(\hat{\mathcal{L}}^n_{i,l})$ versus iteration index l. Each column represents the distribution of $\epsilon(\hat{\mathcal{L}}^n_{i,l}) \,:\, \forall\, n \in \mathcal{N} \setminus \hat{\mathcal{L}}_{i,(l-1)}$. **b**, Standard deviation σ_l of error vectors $\boldsymbol{\epsilon}_i(l)$ versus l. Red lines indicate when the connectivity is completely revealed.

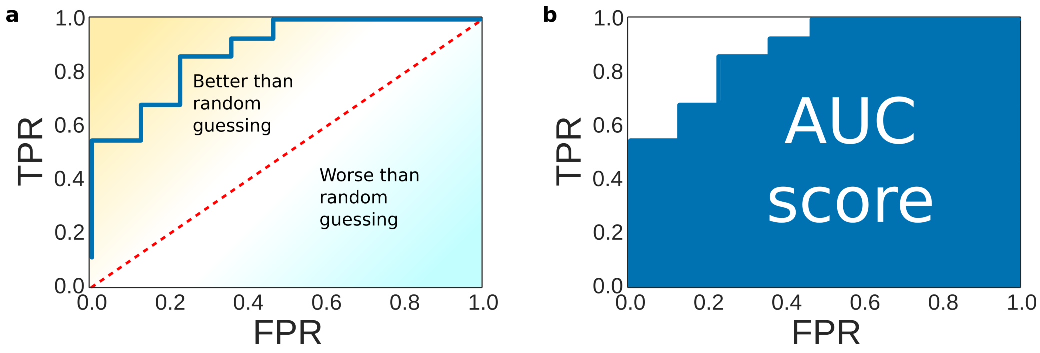

Supplementary Figure 4: **Schematics of ROC curves and AUC scores.** **a**, Generic example of a ROC curve. The ROC space may be divided in two elemental regions separated by the line TPR = FPR. Classifiers above the diagonal line behave better than random-guessing whereas classifiers below the line behave worse than random-guessing. This curve indicates that the classifier correctly predicts true positives while having few false positives. **b**, AUC score for the classifier shown in **a**. The AUC score measures the area under the ROC curve, thus, it reaches a maximum at AUC score = 1 for a perfect classifier.

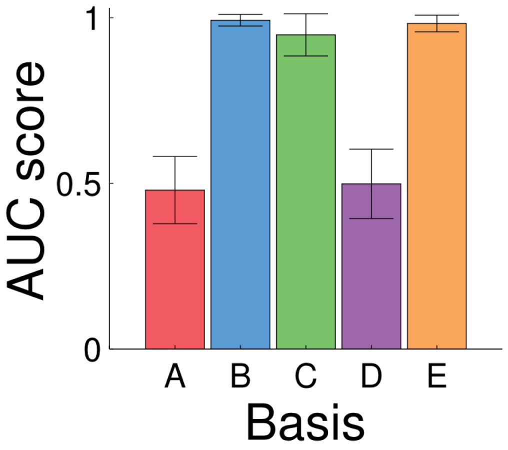

$$A : h_{j,p}(x_j) = x_j^p$$

$$B : h_{ij,p}(x_i, x_j) = (x_j - x_i)^p$$

$$C : h_{ij,p_1p_2}(x_i, x_j) = x_j^{p_1} x_i^{p_2}$$

$$D : h_{j,p}(x_j) = \begin{array}{l} \sin(p(x_j)) \\ \cos(p(x_j)) \end{array}$$

$$E : h_{ij,p}(x_i, x_j) = \begin{array}{l} \sin(p(x_j - x_i)) \\ \cos(p(x_j - x_i)) \end{array}$$

Supplementary Figure 5: **Appropriate basis functions reveal network interactions.** AUC score for reconstruction of networks of $N = 60$ and $n = 20$ Kuramoto-like oscillators (17) employing distinct basis functions, Basis $\in [A, B, C, D, E]$. Choosing a set of basis functions that do not capture the nature of interactions leads to erroneous results.

Parameter	Values
J_0	2.50 mM min^{-1}
k_1	100.00 mM^{-1}min^{-1}
k_2	6.00 mM^{-1}min^{-1}
k_3	16.00 mM^{-1}min^{-1}
k_4	100.00 mM^{-1}min^{-1}
k_5	1.28 min^{-1}
k_6	12.00 mM^{-1}min^{-1}
k	1.80 min^{-1}
κ	13.00 min^{-1}
q	4.00
K_1	0.52 mM
ψ	0.10 mM
N	1.00 mM
A	4.00 mM

Supplementary Table I: Parameters for glycolytic oscillator.

Supplementary Note 1 Algorithm for revealing network interactions ARNI

We decompose units' dynamics into interaction terms with other units in the network as

$$\dot{x}_i \;=\; f_i(\Lambda^i \boldsymbol{x}) \;=\; \sum_{j=1}^{N} \Lambda^i_{jj} g^i_j(x_j) + \sum_{j=1}^{N}\sum_{s=1}^{N} \Lambda^i_{jj}\Lambda^i_{ss} g^i_{js}(x_j, x_s) + \sum_{j=1}^{N}\sum_{s=1}^{N}\sum_{w=1}^{N} \Lambda^i_{jj}\Lambda^i_{ss}\Lambda^i_{ww} g^i_{jsw}(x_j, x_s, x_w) + \ldots \quad (1)$$

where $g^i_j : \mathbb{R} \to \mathbb{R}$, $g^i_{js} : \mathbb{R}^2 \to \mathbb{R}$, $g^i_{jsw} : \mathbb{R}^3 \to \mathbb{R}$ and, in general $g^i_{j_1 j_2 \ldots j_K} : \mathbb{R}^K \to \mathbb{R}$, represent interactions from units j_k to unit i up to the K-th order, such that $k \in \{1, 2, \ldots, K\}$.
Specifically, decomposition (1) separates contributions to units' dynamics arising from different orders, e.g. pairwise and higher order interactions with other units in the system. Thus, it may be employed to infer network connectivity. Here reconstructing the network connectivity implies identifying which interactions in (1) capture the network dynamics the best. So, given that f_i and Λ^i are unknown, functions $g^i_{j_1 j_2 \cdots j_K}$ are not directly accessible. Still, one may expand these functions in terms of basis functions as

$$\dot{x}_i = \sum_{j=1}^{N} \Lambda^i_{jj} \sum_{p=1}^{P_1} c^i_{j,p} h_{j,p}(x_j) + \sum_{j=1}^{N}\sum_{s=1}^{N} \Lambda^i_{jj}\Lambda^i_{ss} \sum_{p=1}^{P_2} c^i_{js,p} h_{js,p}(x_j, x_s) +$$
$$\sum_{j=1}^{N}\sum_{s=1}^{N}\sum_{w=1}^{N} \Lambda^i_{jj}\Lambda^i_{ss}\Lambda^i_{ww} \sum_{p=1}^{P_3} c^i_{jsw,p} h_{jsw,p}(x_j, x_s, x_w) + \ldots, \quad (2)$$

where P_k indicates the number of basis functions employed in the expansion. The rationale behind (2) is to express the unknown functions $g^i_{j_1 j_2 \cdots j_K}$ in terms of linear combinations of simpler and known functions [1].
Hence, defining

$$\boldsymbol{h}_j(x_j) := [h_{j,1}(x_j), h_{j,2}(x_j), \ldots, h_{j,P_1}(x_j)]^{\mathsf{T}} \in \mathbb{R}^{P_1} \quad (3)$$

and $\boldsymbol{c}^i_j := \left[c^i_{j,1}, c^i_{j,2}, \ldots, c^i_{j,P_1}\right] \in \mathbb{R}^{P_1}$, and analogously for all expansions in Supplementary Equation (2), we may rewrite (2) as

$$\dot{x}_i \;=\; \sum_{j=1}^{N} \Lambda^i_{jj} \boldsymbol{c}^i_j \boldsymbol{h}_j(x_j) + \sum_{j=1}^{N}\sum_{s=1}^{N} \Lambda^i_{jj}\Lambda^i_{ss} \boldsymbol{c}^i_{js} \boldsymbol{h}_{js}(x_j, x_s) + \sum_{j=1}^{N}\sum_{s=1}^{N}\sum_{w=1}^{N} \Lambda^i_{jj}\Lambda^i_{ss}\Lambda^i_{ww} \boldsymbol{c}^i_{jsw} \boldsymbol{h}_{jsw}(x_j, x_s, x_w) + \ldots. \quad (4)$$

Now, let us assume that we are given a high dimensional time series of a network $x_{i,m} = x_i(t_m)$ for all $i \in \{1, 2, \ldots, N\}$, where $t_m = m\Delta t + t_0$ is the sampling time for $m \in \{1, 2, \ldots, M\}$ and $\dot{x}_{i,m} = \dot{x}_i(t_m)$ is the time derivative of i computed (e.g. through finite differences) at t_m. We may rewrite Supplementary Equation (4) as

$$\dot{\boldsymbol{x}}_i = \sum_{j=1}^{N} \Lambda^i_{jj} \boldsymbol{c}^i_j H_j + \sum_{j=1}^{N}\sum_{s=1}^{N} \Lambda^i_{jj}\Lambda^i_{ss} \boldsymbol{c}^i_{js} H_{js} + \sum_{j=1}^{N}\sum_{s=1}^{N}\sum_{w=1}^{N} \Lambda^i_{jj}\Lambda^i_{ss}\Lambda^i_{ww} \boldsymbol{c}^i_{jsw} H_{jsw} + \ldots, \quad (5)$$

where $\dot{\boldsymbol{x}}_i := [\dot{x}_{i,1}, \dot{x}_{i,2}, \ldots, \dot{x}_{i,M}] \in \mathbb{R}^M$,

$$H_j := [\boldsymbol{h}_j(x_{j,1}), \boldsymbol{h}_j(x_{j,2}), \ldots, \boldsymbol{h}_j(x_{j,M})] \in \mathbb{R}^{P_1 \times M}, \quad (6)$$

and matrices H_{js} and H_{jsw} are constructed in a similar manner to (6).
The reconstruction problem then becomes identifying the non-zero interaction terms in Supplementary Equation (5) that best capture the unit's dynamics. Yet, the vector of coefficients $\boldsymbol{c}^i_j, \boldsymbol{c}^i_{js}, \boldsymbol{c}^i_{jsw}$ (and so on) are unknown, thereby, hindering the retrieval of Λ^i from (5).
Solving linear systems (5) consist of finding *block structures for* $\boldsymbol{c}^i$. These structured solutions are composed by blocks $\boldsymbol{c}^i_s$ of non-zero entries (representing the non-zero interactions acting on unit i) distributed along $\boldsymbol{c}^i$, cf. **1**. However, such solutions cannot be retrieved in standard manners (e.g. Moore-Penrose pseudo-inversion or minimization of L_1-norm, cf. [2]). Thus, solving this kind of systems requires more sophisticated methods.
Specifically, given a linear system

$$\boldsymbol{y} = \boldsymbol{c}D, \quad (7)$$

where $\boldsymbol{y} \in \mathbb{R}^{1\times M}$ is a vector of M measurements and $D \in \mathbb{R}^{Nd\times M}$ is a matrix divided into N matrix blocks $D_j \in \mathbb{R}^{d\times M}$ for all $j \in \{1, 2, \ldots, N\}$, we may define a structured solution in blocks for $\boldsymbol{c}$ as [3, 4, 5, 6, 7]

$$\boldsymbol{c} = \big[\underbrace{c_1, c_2, \ldots, c_d}_{\boldsymbol{c}_1}, \underbrace{c_{d+1}, c_{d+2}, \ldots, c_{2d}}_{\boldsymbol{c}_2}, \ldots, \underbrace{c_{(N-1)d+1}, c_{(N-1)d+2}, \ldots, c_{Nd}}_{\boldsymbol{c}_N}\big], \tag{8}$$

where $\boldsymbol{c}_j \in \mathbb{R}^d$ for all $j \in \{1, 2, \ldots, N\}$ represent the blocks.
Recent efforts on solving systems (7) subjected to solutions (8) in blocks have been mainly focused on *block sparse*[1] *solutions on under-determined systems* ($Nd \gg M$) [3, 4, 5, 6, 7], cf. **1**. Such approaches (e.g. group lasso [3], sparse regression using mixed norms [5], block orthogonal matching pursuit [6]) take advantage of the indeterminacy of systems (7) to iteratively construct a block sparse solution from a family of solutions for (7) employing specific heuristics (e.g. minimizing $\|\boldsymbol{c}_j\|_2$ for $j \in \{1, 2, \ldots, N\}$ while maximizing the number of zero blocks [4]).
Here, we developed the *Algorithm for Revealing Network Interactions* (ARNI) that reverse-engineers the non-zero interactions in Supplementary Equation (5) while solely relying on measured time series of $\boldsymbol{x}$. Specifically, ARNI: (i) constructs function spaces for each interaction using the known basis functions and measured time series, (ii) finds the best suited set of interactions that reproduces the measured $\dot{x}_i$ under a squared error-minimization scheme and (iii) returns the explicit connections Λ^i of the unit. Formally, ARNI is based on the Block Orthogonal Least Squares (BOLS) [9] algorithm.
Opposite to methods described in [3, 4, 5, 6, 7], ARNI recovers connections by minimizing orthogonal projections on *sequentially-enlarged* function spaces generated by the basis functions, cf. Supplementary Figures **2** and **3**. Thus, ARNI iteratively finds the composite function space that captures the recorded dynamics the best.
Conceptually, the algorithm consists of the following steps:

1. Select a generic model for interactions by choosing orders K, P_1, P_2, P_3 and so on, in (2).

2. Select models for basis functions $h_{j,p}(x_j)$, $h_{js,p}(x_j, x_s)$, $h_{jsw,p}(x_j, x_s, x_w)$ and so on, cf. Supplementary Note 6.

3. Compute vectors $\dot{\boldsymbol{x}}_i = [\dot{x}_{i,1}, \dot{x}_{i,2}, \ldots, \dot{x}_{i,M}] \in \mathbb{R}^M$ for all i and construct the set of matrices $\mathcal{H}$ up to chosen K, where $\mathcal{H}$ is defined as
$$\mathcal{H} := \{H_q | q = 1, 2, \ldots, Q\}, \tag{9}$$
where $H_{N+j} := H_{jj}$, $H_{N+N^2+j} := H_{jjj}$ and so on, and $Q = \sum_{k=1}^{K} N^k$.

4. Define the set $\mathcal{N} := \{1, 2, \ldots, S\}$ that contains all s indices of $\mathcal{H}$.

5. Define the set of incoming interactions of unit i at the l-th iteration as $\hat{\mathcal{L}}_{i,l}$, and initialize $\hat{\mathcal{L}}_{i0} = \varnothing$.

6. Compute $\forall\, n \in \mathcal{N} \setminus \hat{\mathcal{L}}_{i,(l-1)}$ the projection-error function
$$\epsilon\left(\hat{\mathcal{L}}^n_{i,l}\right) := \sigma\left(\dot{\boldsymbol{x}}_i - H^{\dagger}_{\hat{\mathcal{L}}^n_{i,l}} H_{\hat{\mathcal{L}}^n_{i,l}} \dot{\boldsymbol{x}}_i\right), \tag{10}$$
where $\sigma(\boldsymbol{y})$ stands for the standard deviation of entries of $\boldsymbol{y}$, $\dagger$ indicates the Moore-Penrose pseudo-inverse, $\hat{\mathcal{L}}^n_{i,l} = \hat{\mathcal{L}}_{i,(l-1)} \cup \{n\}$ and
$$H_{\hat{\mathcal{L}}^n_{i,l}} := \left[H_{j_1}, H_{j_2}, \ldots, H_{j_{l-1}}, H_n\right]^{\mathsf{T}} \in \mathbb{R}^{lP\times M}, \tag{11}$$
where $j_p \in \hat{\mathcal{L}}_{i,(l-1)}$ and $p = \{1, 2, \ldots, l-1\}$.

7. Store results in $\boldsymbol{\epsilon}_i(l) \in \mathbb{R}^L$ where $[\boldsymbol{\epsilon}_i(l)]_r = \epsilon\left(\hat{\mathcal{L}}^{n_r}_{i,l}\right)$, $n_r \in \mathcal{N} \setminus \hat{\mathcal{L}}_{i,(l-1)}$, $r \in \{1, 2, \ldots, L\}$ and $L = \left|\mathcal{N} \setminus \hat{\mathcal{L}}_{i,(l-1)}\right|$.

8. If the standard deviation $\sigma_l := \sigma(\boldsymbol{\epsilon}_i(l)) \leq \theta$, STOP algorithm and RETURN $\hat{\mathcal{L}}_{i,(l-1)}$ as set of incoming interactions.

9. Else, UPDATE $\hat{\mathcal{L}}_{i,l} = \hat{\mathcal{L}}_{i,(l-1)} \cup \{n^*\}$ where
$$n^* = \arg \min_{n\in\mathcal{N}\setminus\hat{\mathcal{L}}_{i,(l-1)}} \left(\epsilon\left(\hat{\mathcal{L}}^n_{i,l}\right)\right) \tag{12}$$
and REPEAT from steps 6 and 7 until the condition in step 8 is satisfied or $\mathcal{N} \setminus \hat{\mathcal{L}}_{i,(l-1)} = \varnothing$.

[1] In the weak definition we follow in this work, a sparse vector consist of an array containing only few entries different from zero [3, 8, 4, 5, 6, 7].

The projection-error function in step 6 differs from the cost function introduced in the main manuscript. Also, for the condition $\mathcal{N} \setminus \hat{\mathcal{L}}_{i,(l-1)} = \varnothing$, the algorithm has a complexity of $\mathcal{O}\left(Q^2\right)$.

Supplementary Note 2 Summary of Scaling Behavior

In what follows, we briefly summarize how the approach scales with respect to different intrinsic features of the networked systems, specifically, the system size, the number of incoming connections per unit, and the noise level in the time series. We also provide an explanation about a key aspect of the algorithmic complexity of our approach. As shown in the main manuscript, the number $M_{0.95}$ of required measurements to achieve reconstructions of AUC > 0.95 scales ...

- sublinearly, likely logarithmically $\sim log(N)$ (see Figure 3a in main manuscript), with number N of dynamical variables in the network (that equals the number of units if they exhibit one-dimensional dynamics). Similar results have been reported in [10, 2].
- linearly,$\sim n_i$ (see Figure 3b in main manuscript), with the number n_i of incoming connections per unit.
- supralinearly (see Figure 3c in main manuscript) with the noise strength η.

Concerning the algorithmic complexity of our approach, if Q is the total number of proposed expansions in the right hand side of Supplementary Equation (5), it follows that the total number of projections for the worst-case condition $\mathcal{N} \setminus \hat{\mathcal{L}}_{i,(l-1)} = \varnothing$ is given by

$$\# \text{ Projections} = \underbrace{Q}_{\text{1st iter.}} + \underbrace{(Q-1)}_{\text{2nd iter.}} + \underbrace{(Q-2)}_{\text{3rd iter.}} + \ldots + \underbrace{1}_{Q\text{-th iter.}} = \frac{Q(1+Q)}{2} \sim \mathcal{O}\left(Q^2\right). \tag{13}$$

Therefore, in the worst case where all Q expansions are needed to approximate the right hand side of Supplementary Equation (5) or the threshold θ is too small, our algorithm has a complexity of $\mathcal{O}\left(Q^2\right)$.

Supplementary Note 3 Quantifying quality of reconstruction

Our reconstruction problem may be seen as a binary classification problem where one has to identify whether network interactions are active or not. Thus, if an *interaction is active* and it is *classified as active*, it is counted as a *true positive*, yet if it is *classified as inactive*, it is counted as a *false negative*. Conversely, if the *interaction is inactive* and it is *classified as inactive*, it is counted as a *true negative*, but if it is *classified as active*, it is counted as a *false positive*.

True positives and false positives rates

We measure the predictive power of our approach using the *true positives* (TPR) and *false positives* (FPR) *rates*. Basically, the TPR quantifies the fraction of active interactions which were *correctly identified as active* from all active interactions (actual interactions) present in the dynamics. Analogously, the FPR quantifies the fraction of inactive interactions which were *incorrectly identified as active* from all inactive interactions available. Both quantities are defined as

$$\text{TPR} = \frac{\text{True positives}}{\text{Total number of positives}}, \tag{14}$$

$$\text{FPR} = \frac{\text{False positives}}{\text{Total number of negatives}}. \tag{15}$$

Receiver Operating Characteristics (ROC) curve

A ROC curve is a two-dimensional graph in which TPR is plotted against FPR. Its purpose is to depict the trade-off between *true positives* and *false positives* of a given classifier [11].
Depending on its internal parameters, the classifier may yield better or worse results. Thus, ROC curves provide a graphical way to assess the predictive power of such classifiers with respect to their internal parameters, cf. **4**a. Ideally, one seeks to have points in the ROC space as close to the upper-left corner as possible. Points there

represent classifiers with high TPR and low FPR. Thus, classifiers with ROC-scores close to the upper left corner are more likely to correctly predict true positives and those close to the diagonal, given that they are applied to a test data set that has similar dynamical properties (stationarity) and is comparable in length to the data set which was used to evaluate the ROC score.
The diagonal line TPR = FPR represents a random-guessing classifier, cf. **4**a. Thus, any classifier that appears below this line performs worse than random-guessing. Conversely, any classifier that appears above performs better than random-guessing.

Area Under the Curve (AUC) score

Still, one may want to characterize the predictive power of a classifier in terms of a single scalar variable rather than interpreting a two-dimensional graph. A broadly used method is to measure the Area Under the ROC Curve, also known as the AUC score [11]. As its name indicates, the AUC score quantifies the area below the ROC curve of a given classifier, and thereby, AUC score $\in [0, 1]$, cf. **4**b.
The AUC score provides an easy way to compare the predictive power of different classifiers. The higher the AUC score of a classifier is, the higher its predictive power. Also, the AUC score is equivalent to the probability that the classifier will rank (in a list of probable interactions) a randomly-selected *active interaction* higher than a randomly-selected *inactive interaction* [11].
In our implementation, we track the evolution of the minimal projection-error $\min_{n \in \mathcal{N}} \left(\epsilon \left(\hat{\mathcal{L}}^{n}_{i,l} \right) \right)$, for each l-iteration, Supplementary Figure **3**. Then, we compute the ROC curve by (i) systematically varying a threshold β on the sequence of minimal projection-errors, and (ii) calculating the TPR and FPR for each β. In particular, given a specific β, we consider as correct interactions those inferred in all iterations whose minimal projection-error is above β. Finally, we compute the AUC score of the resulting curve.

Supplementary Note 4 Random network model systems

To test our framework, we generated high dimensional time series simulating directionally coupled networks of dynamical systems. The resulting time series were used as inputs for reconstructing network structural connections by means of ARNI. To mimic real-world recordings, we sampled time series with sampling rates of $\Delta t = 1$ for all simulations (except where stated). Nonetheless, we note that simulations were computed with time steps $\delta t \ll \Delta t$.

Ensembles inspired by gene regulatory circuits

To test the predictive power of our approach on systems mimicking gene regulation, we simulated networks of dynamical systems having Michaelis-Menten kinetics [12, 13]

$$\dot{x}_i = -x_i + \frac{1}{n_i} \sum_{j=1}^{N} J_{ij} \frac{x_j}{1 + x_j}, \tag{16}$$

having n_i randomly-selected incoming connections per node. Here, the entries of J_{ij} of $J \in \mathbb{R}^{N \times N}$ are given by $J = R \odot A$, where $\odot$ stands for entry-wise-matrix product, $A \in \{0, 1\}^{N \times N}$ is a randomly generated adjacency matrix with a fixed number n_i of non-zero entries per row and $R \in \mathbb{R}^{N \times N}$ with R_{ij} randomly drawn from a uniform distribution $R_{ij} \in [1, 5]$.

Networks and Hypernetworks of Kuramoto-like oscillators

To introduce functional complexity in the coupling functions, we selected a model of Kuramoto-like oscillators with coupling functions having two Fourier modes [14]

$$\dot{x}_i = \omega_i + \frac{1}{n_i} \sum_{j=1}^{N} J_{ij} \left[\sin\left(x_j - x_i - 1.05\right) + 0.33 \sin\left(2\left(x_j - x_i\right)\right) \right] + \xi_i, \tag{17}$$

having n_i randomly-selected incoming connections per node. Here, the entries of J_{ij} of $J \in \mathbb{R}^{N \times N}$ are given by $J = R \odot A$, $A \in \{0, 1\}^{N \times N}$ is a randomly generated adjacency matrix with a fixed number n_i of non-zero entries per row and $R \in \mathbb{R}^{N \times N}$ with R_{ij} randomly drawn from a uniform distribution $R_{ij} \in [0.5, 1]$. The natural frequencies

ω_i are randomly selected from a uniform distribution $\omega_i \in [-2, 2]$ and ξ_i represents external white noise affecting the dynamics of unit i.
To characterize the selectivity of ARNI against network and hypernetwork interactions, we simulated extended models of (17) where

$$\dot{x}_i = \omega_i + \frac{1}{n_i} \sum_{j=1}^{N} \sum_{k=1}^{N} E^i_{jk} \left[\sin\left(x_j - x_k - 1.05\right) + 0.33 \sin\left(2\left(x_j - x_k\right)\right)\right] + \xi_i, \tag{18}$$

with n_i randomly-selected incoming interactions per node. Differently to (17), here we introduce the second order interaction matrix $E^i \in \mathbb{R}^{N \times N}$ for all $i = \{1, 2, \ldots, N\}$. Specifically, the element E^i_{jk} weighs how units j and k directly influence unit i together. Thus, it follows that the elements E^i_{ik} for all $k = \{1, 2, \ldots, N\}$ represent regular network interactions. The entries of E^i are set by $E^i = R \odot C$, where $C \in \{0, 1\}^{N \times N}$ is a randomly generated matrix with a fixed number n_i of 1's across the matrix. The R_{ij} entries of $R \in \mathbb{R}^{N \times N}$ are randomly drawn from a uniform distribution $R_{ij} \in [0.5, 1]$.

Networks of Rössler oscillators

To test the robustness of our approach against chaotic dynamics, we simulated networks of coupled Rössler oscillators [15]. The dynamics of each oscillator $\boldsymbol{x}_i = \left[x^1_i, x^2_i, x^3_i\right] \in \mathbb{R}^3$ is set by three differential equations

$$\dot{x}^1_i = -x^2_i - x^3_i + \frac{1}{n_i} \sum_{j=1}^{N} J_{ij} \sin(x^1_j) + \xi^1_i, \tag{19}$$

$$\dot{x}^2_i = x^1_i + 0.1 x^2_i + \xi^2_i, \tag{20}$$

$$\dot{x}^3_i = 0.1 + x^3_i \left(x^1_i - 18.0\right) + \xi^3_i, \tag{21}$$

where n_i is the number of incoming connections to unit i. As before, the entries of J are given by $J = R \odot A$, where $R_{ij} \in [0.5, 1]$. Similarly, ξ^k_i with $k \in \{1, 2, 3\}$ represent external noisy signals acting on the unit's components.

Supplementary Note 5 Biological model systems

To test performance on biological model systems, we simulated the glycolytic oscillator in yeast [16] and circadian clock in *Drosophila* [17] and inferred their interactions from their collective dynamics.

Glycolytic oscillator model

Glycolytic oscillations is one of the best-studied examples for oscillations at the cellular level. All its factors oscillate at different phases with a shared relatively short period. In yeast cells, these oscillations were also shown to synchronize across cell population [18]. Here we use a single-cell model for anaerobic glycolytic oscillations in yeast, containing the influx of glucose and outflux of pyruvate and/or acetaldehyde, resulting in coupled ODE equations for seven species [16]

$$\dot{S}_1 = J_0 - \frac{k_1 S_1 S_6}{1 + (S_6/K_1)^q} \tag{22}$$

$$\dot{S}_2 = 2\frac{k_1 S_1 S_6}{1 + (S_6/K_1)^q} - k_2 S_2 (N - S_5) - k_6 S_2 S_5 \tag{23}$$

$$\dot{S}_3 = k_2 S_2 (N - S_5) - k_3 S_3 (A - S_6) \tag{24}$$

$$\dot{S}_4 = k_3 S_3 (A - S_6) - k_4 S_4 S_5 - \kappa (S_4 - S_7) \tag{25}$$

$$\dot{S}_5 = k_2 S_2 (N - S_5) - k_4 S_4 S_5 - k_6 S_2 S_5 \tag{26}$$

$$\dot{S}_6 = -2\frac{k_1 S_1 S_6}{1 + (S_6/K_1)^q} + 2 k_3 S_3 (A - S_6) - k_5 S_6 \tag{27}$$

$$\dot{S}_7 = \psi \kappa (S_4 - S_7) - k S_7 \tag{28}$$

where S_1 represents glucose, S_2 represents glyceraldehydes-3-phosphate and dihydroxyacetone phosphate pool, S_2 represents 1,3-bisphosphoglycerate, S_2 represents cytosolic pyruvate and acetaldehyde pool, S_2 represents NADH, S_2 represents ATP, and S_7 represents extracellular pyruvate and acetaldehyde pool. Parameters were set according to [19] and are shown in Supplementary Table **I**. Initial conditions were randomly selected from the uniform distribution $S_i(0) \in [1.2, 2.0]$ and employed $\Delta T = 0.01$ min.

Circadian Clock

Circadian clocks underly the response to the day-night cycle. Specifically, we simulated a model of the circadian clock in *Drosophila* [17], where oscillations are driven by a negative feedback between *per* and *tim* genes, which code for PER and TIM proteins, and the PER-TIM complex. The rate equations for the 10-species circadian clock are:

$$\dot{M}_p = v_{sP}\frac{K_{IP}^n}{K_{IP}^n + C_N^n} - v_{mP}\frac{M_P}{K_{mP} + M_P} - k_d M_P \tag{29}$$

$$\dot{P}_0 = k_{sP}M_P - V_{1p}\frac{P_0}{K_{1P} + P_0} + V_{2p}\frac{P_1}{K_{2P} + P_1} - k_d P_0 \tag{30}$$

$$\dot{P}_1 = V_{1p}\frac{P_0}{K_{1P} + P_0} - V_{2p}\frac{P_1}{K_{2P} + P_1} - V_{3p}\frac{P_1}{K_{3P} + P_1} + V_{4p}\frac{P_2}{K_{4P} + P_2} - k_d P_1 \tag{31}$$

$$\dot{P}_2 = V_{3P}\frac{P_1}{K_{3P} + P_1} - V_{4p}\frac{P_2}{K_{4P} + P_2} - k_3 P_2 T_2 + k_4 C - v_{dP}\frac{P_2}{K_{dP} + P_2} - k_d P_2 \tag{32}$$

$$\dot{M}_T = v_{sT}\frac{K_{IT}^n}{K_{IT}^n + C_N^n} - v_{mT}\frac{M_T}{K_{mT} + M_T} - k_d M_T \tag{33}$$

$$\dot{T}_0 = k_{sT}M_T - V_{1T}\frac{T_0}{K_{1T} + T_0} + V_{2T}\frac{T_1}{K_{2T} + T_1} - k_d T_o \tag{34}$$

$$\dot{T}_1 = V_{1T}\frac{T_0}{K_{1T} + T_0} - V_{2T}\frac{T_1}{K_{2T} + T_1} - V_{3T}\frac{T_1}{K_{3T} + T_1} + V_{4T}\frac{T_2}{K_{4T} + T_2} - k_d T_1 \tag{35}$$

$$\dot{T}_2 = V_{3T}\frac{T_1}{K_{3T} + T_1} - V_{4T}\frac{T_2}{K_{4T} + T_2} - k_3 P_2 T_2 + k_4 C - V_{dT}\frac{T_2}{K_{dT} + T_2} - k_d T_2 \tag{36}$$

$$\dot{C} = k_3 P_2 T_2 - k_4 C - k_1 C - k_2 C_N - k_{dC} C \tag{37}$$

$$\dot{C}_N = k_1 C - k_2 C_N - k_{dN} C_N \tag{38}$$

where M_T and M_P are *tim* and *per* mRNAs, respectively. T_0, T_1 and T_2 are forms of TIM protein, P_0, P_1 and P_2 are forms of PER protein, and C and C_N are forms of PER-TIM complex. The parameter values used for the simulations are based on [17]. The total levels of PER and TIM proteins, P_t and T_t, respectively, are given by:

$$P_t = P_0 + P_1 + P_2 + C + C_N \tag{39}$$

$$T_t = T_0 + T_1 + T_2 + C + C_N \tag{40}$$

Supplementary Note 6 Choosing a proper family of basis functions

Selecting an appropriate class of basis functions to represent the network interactions is vital step of our approach. Choosing basis functions that capture the intrinsic nature of interactions (e.g. single $h(x_i)$, pairwise $h(x_i, x_j)$, triplet $h(x_i, x_j, x_w)$, and so on) will lead to optimal results. However, finding an appropriate class of basis functions should not be confused with finding a specific set of basis functions. While the former implies finding basis functions of correct order $k \in \{1, 2, \ldots, K\}$, Supplementary Figure **5**, the latter implies finding a unique set of basis functions capable of representing the network dynamics.

Reconstruction of networks of Kuramoto-like oscillators (17) using different sets of basis functions reveal that employing bases resembling the intrinsic nature of interactions (e.g. pairwise) lead to optimal results, Supplementary Figure **5**. The rationale is that expansions in basis functions seek to span functional spaces, thus, functions involving x_i and x_j together, $h(x_i, x_j)$, are more suitable to represent the dependencies induced by pairwise interactions in (17) than functions just involving single interactions, $h(x_i)$ and $h(x_j)$.

Supplementary References

[1] Hastie, T., Tibshirani, R., and Friedman, J. *The Elements of Statistical Learning*. Springer Series in Statistics. Springer New York, New York, NY, (2009).

[2] Timme, M. and Casadiego, J. *J. Phys. A Math. Theor.* **47**(34), 343001 aug (2014).

[3] Yuan, M. and Lin, Y. *J. R. Stat. Soc. Ser. B Stat. Methodol.* **68**(1), 49–67 feb (2006).

[4] Eldar, Y. C. and Mishali, M. *IEEE Trans. Inf. Theory* **55**(11), 5302–5316 nov (2009).

[5] Kowalski, M. *Appl. Comput. Harmon. Anal.* **27**(3), 303–324 nov (2009).

[6] Eldar, Y. C., Kuppinger, P., and Bölcskei, H. *IEEE Trans. Signal Process.* **58**(6), 3042–3054 jun (2010).

[7] Duarte, M. F. and Eldar, Y. C. *IEEE Trans. Signal Process.* **59**(9), 4053–4085 sep (2011).

[8] Napoletani, D. and Sauer, T. D. *Phys. Rev. E* **77**(2), 26103 feb (2008).

[9] Majumdar, A. and Ward, R. K. *Can. J. Electr. Comput. Eng.* **34**(4), 136–144 (2009).

[10] Timme, M. *Phys. Rev. Lett.* **98**(22), 224101 (2007).

[11] Fawcett, T. *Pattern Recognit. Lett.* **27**(8), 861–874 (2006).

[12] Karlebach, G. and Shamir, R. *Nat. Rev. Mol. Cell Biol.* **9**(10), 770–780 oct (2008).

[13] Barzel, B. and Barabási, A.-L. *Nat. Biotechnol.* **31**(8), 720–5 aug (2013).

[14] Hansel, D., Mato, G., and Meunier, C. *Phys. Rev. E* **48**(5), 3470–3477 nov (1993).

[15] Rössler, O. E. *Phys. Lett. A* **57**(5), 397–398 jan (1976).

[16] Wolf, J. and Heinrich, R. *Biochem. J.* **345**, 321–34 jan (2000).

[17] Leloup, J.-C. and Goldbeter, A. *J. Theor. Biol.* **198**(3), 445–459 jun (1999).

[18] Richard, P. *FEMS Microbiol. Rev.* **27**(4), 547–557 oct (2003).

[19] Daniels, B. C. and Nemenman, I. *Nat. Commun.* **6**, 38 (2014).
